# Discontinuation of affirmative action: Consequences for black educational equity, neurosurgical residency, and medical diversity, with consideration of potential adversity as a new path forward

**DOI:** 10.1016/j.wnsx.2024.100339

**Published:** 2024-02-24

**Authors:** Albert Alan, Michelle Ennabe, Abdulmuizz Sulaiman, Martin Weinand

**Affiliations:** a Department of Neurosurgery, University of Arizona, Tucson, AZ, USA; b University of Arizona College of Medicine, Tucson, AZ, USA; c Global Neurosurgical Alliance, Tucson, AZ, USA; d University of Arizona College of Medicine, Phoenix, AZ, USA; e College of Health Sciences, University of Ilorin, Kwara State, Nigeria

**Keywords:** Underrepresentation, Affirmative action, Neurosurgery admissions, Racial concordance, ALDC admissions criteria, Holistic admission approaches

## Abstract

**Background:**

The underrepresentation of the Black community in neurosurgery is concerning, especially given projections that racial minorities will become the majority in the U.S. by 2044. Yet, despite these forecasts, Black candidates make up less than 4% of those in neurosurgical training programs. The recent Supreme Court decision to end Affirmative Action underscores the urgency of addressing this disparity. This research delves into the implications of eliminating Affirmative Action on neurosurgery admissions and residencies.

**Methods:**

A comprehensive literature search was performed using PubMed, OVID Embase, and OVID Medline, employing the keywords “Black”, “Neurosurgery”, and “Residency”. The Maslow Adversity Index (MAI) was created to integrate adversity as a factor in neurosurgery residency evaluation.

**Results:**

After Affirmative Action, Black college enrollment increased, peaking at 36% by 2020. However, Black medical students remain underrepresented in neurosurgery residencies. ALDC (Athletes, Legacies, Dean’s List, Children of faculty/staff) admissions criteria favor White students. Furthermore, studies have highlighted the beneficial impacts of racial concordance on patient outcomes. The end of Affirmative Action necessitates new diversity strategies in admissions. A points-based assessment, inspired by Maslow's hierarchy, recognizes adversities faced by underrepresented applicants which could help residency programs enhance diversity, inclusivity, and equity in selection.

**Conclusion:**

Despite the growth in Black college attendance, disparities persist in specialized medical fields like neurosurgery. The end to Affirmative Action policies might exacerbate these disparities. Embracing holistic admission approaches, rooted in Maslow's hierarchy. This consideration is key for inclusive representation, impacting education, professions, and health outcomes.

## 1 Introduction

The Black community has consistently grappled with challenges of inequality, particularly the lack of equitable representation in medical subspecialties such as neurosurgery.^18^ This deficiency in representation has persisted over time, emphasizing the pressing need for universities to address and rectify this disparity. In the wake of the 2020 Black Lives Matter movement, academic and medical institutions globally initiated introspective evaluations, leading to the restructuring of their practices to better embody principles of equity and anti-racism, as epitomized by initiatives.^7^ However, despite this progressive global stance, a pivotal decision on June 29, 2023, by the Supreme Court signaled the conclusion of Affirmative Action-policies and legal measures designed to redress past and prevent future discrimination, ensuring that individuals are not denied opportunities based on race, ethnicity, religion, nationality, age, gender, or disability. This verdict emerged from lawsuits filed against Harvard University and the University of North Carolina, challenging their race-conscious admission strategies.^13^ Historically, institutions have judiciously weighed various factors, including race, in their admissions processes.^13^ The intent of this paper is to elucidate the broader implications of this decision and offer a strategic framework to guide medical school admissions and neurosurgical residency programs in supporting historically marginalized populations.

Looking ahead to 2044, projections suggest that racial minority groups will account for a majority of the U.S. population.^12^ Despite these demographic changes, disparities persist, especially in specialized medical fields such as neurosurgery. Studies indicate a notable interest in neurosurgery among Black medical students.^3^ However, these Black medical student candidates secure neurosurgical residencies at rates lower than their non-Black peers.^14^ Furthermore, Black residents constitute less than 4% of participants in neurosurgical training programs.^3^ Concerns arise that removing or reducing the emphasis on race and ethnicity in admissions could impact the efforts of medical institutions in maintaining diverse student cohorts and might influence the pool of minority candidates considering advanced medical training.^13^

This study is grounded in an exhaustive literature review examining the disparities faced by Black individuals in the field of neurosurgery. The findings reveal inequality that disproportionately affects Black medical school candidates interested in this specialization. Prompted by the discussions surrounding the elimination of Affirmative Action, this research endeavors to assess its potential impact. A comprehensive analysis of relevant sources underscores the existing challenges encountered by Black medical students aspiring to pursue neurosurgery. As such, the primary aim of this investigation is to harness insights from the literature to guide neurosurgery residency committees and medical school admission committees in ways to effectively support Black students in their academic and professional pursuits.

## 2 Methods

A literature review was conducted to investigate the current corpus of research pertaining to Black residents in neurosurgery. This thorough investigation involved searching PubMed, OVID Embase, and OVID Medline databases. The search duration encompassed the period from the establishment of each individual database up until September 2023. The primary search strategy employed the keywords “Black” “Neurosurgery” “Residency” to retrieve relevant studies and articles. The term “Black” was selected to inclusively represent individuals with darker skin tones, acknowledging the diversity within this demographic that encompasses a global population beyond the singular identity of African American. The central aim of this study is to critically analyze the experiences and the systemic challenges faced by the Black community, specifically pertaining to their underrepresentation in neurosurgery residency programs.

Additional articles were selected outside the database search criteria, along with supplementary searches were carried out on governmental and nonprofit websites. These sources included The White House, the Postsecondary National Policy Institute, National Bureau of Economic Research, and the Association of American Medical Colleges.

**Fig. 1:**
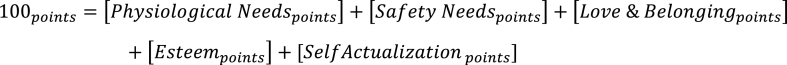
Illustration of the ‘Maslow adversity Index (MAI)', a quantitative measure designed for evaluating adversity in neurosurgery applicants.

Moreover, a computational psychological model was developed based on Maslow's Hierarchy of Needs. This model incorporates an equation, as seen in Fig. 1, specifically designed to measure diversity scores. The equation is as follows.

### 2.1 Comprehensive residency adversity score

$$100_{points}=\left[{Physiological\;Needs}_{points}\right]+\left[{Safety\;Needs}_{points}\right]+\left[{Love\;\&\;Belonging}_{points}\right]+\left[{Esteem}_{points}\right]+\left[{SelfActualization}_{\;points}\right]$$

The MAI equation quantitatively evaluates the fulfillment of each level of needs, assigning weighted values to different aspects of Maslow's hierarchy. The model thus provides a numerical score that reflects the diversity in the satisfaction of these needs, enabling a more nuanced analysis of psychological states in varying contexts.

## 3 Results

**Fig. 2:**
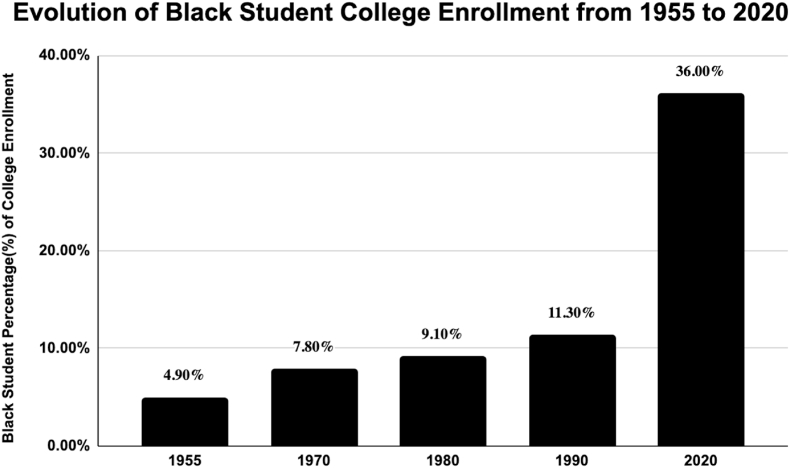
**Evolution of Black Student College Enrollment from 1955 to 2020** *A chart depicting the percentage of Black students entry into college over decades. This graph was modified from: “Affirmative Action: History and Rationale” and “Black students in Higher Education”*.^1^^,^^5^

**Fig. 3:**
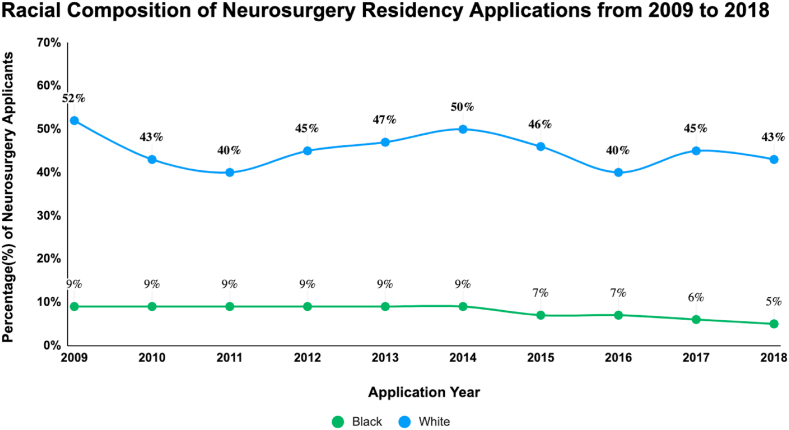
Racial Composition of Neurosurgery Residency Applications from 2009 to 2018 *A chart illustrating the percentage of Black and White applicants to neurosurgery residency programs, highlighting disparities over a decade. This graph was modified from “Diversity in Neurosurgery: Trends in Gender and Racial/Ethnic Representation Among Applicants and Residents from U.S. Neurological Surgery Residency Programs”*^12^

**Fig. 4:**
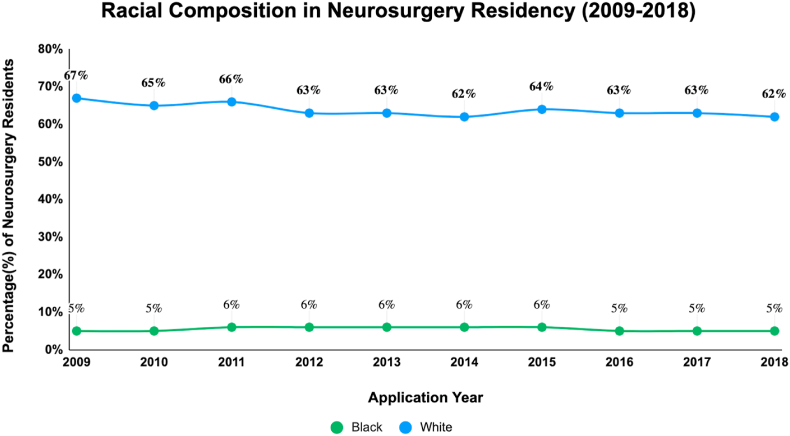
**Racial Composition in Neurosurgery Residency (2009–2018)** A graph showing the consistent underrepresentation of Black residents in neurosurgery residencies compared to White residents across a decade. This graph was modified from “Diversity in Neurosurgery: Trends in Gender and Racial/Ethnic Representation Among Applicants and Residents from U.S. Neurological Surgery Residency Programs”.^12^

Table 1 compiles data from various academic studies focusing on Black academic inequality across college, medical school and residency. The initial reference derives from “AFFIRMATIVE ACTION: HISTORY AND RATIONALE” and “Black Students in Higher Education".^1^^,^^5^ As depicted in Fig. 2, following the inception of affirmative action, there was a noticeable surge in Black college enrollment from the 1970s, peaking at 36% by 2020.^1^^,^^5^ Arcidiacono et al's research points out that White ALDC students enjoy specific benefits during the admissions process. Legacy students see a fivefold spike in admission rates, dean's list students experience a sevenfold rise, and recruited athletes are highly likely to be accepted.^2^ Concurrently, Bhutta et al's 2019 SCF emphasizes that significant racial wealth gaps persist, with White households holding roughly eight times the wealth of Black households.^4^ Furthermore, Maqsood et al, 2021 also provided insights. In the broader context, the US Census Bureau data underscores that minority groups, like the Black community, are undergoing swift population growth.^16^ As of 2015, nearly half of American children below 18 years are part of ethnic minorities, and the foreign-born population more than doubled from 1990, constituting a third of the overall population increase.^16^ Gabriel et al, 2021, elaborates on changes that will be happening by 2044 in the United States where racial minorities is projected to soon become the majority.^12^ Fig. 3, Fig. 4, adapted from the work of Gabriel et al, present an analysis of racial disparities in neurosurgery applications and residencies over a nine-year period from 2009 to 2018. Fig. 3 illustrates a significant disproportion in the number of White versus Black medical students applying for neurosurgery. Specifically, the data reveal a consistently higher number of White applicants compared to their Black counterparts. Furthermore, there is a noticeable downward trend in Black medical student applicants: from 9% in 2009 to 7% in 2015, and further declining to 5% in 2018. In contrast, the proportion of White applicants shows more fluctuation: 53% in 2009, decreasing to 46% in 2015, and then to 43% in 2018. Fig. 4 focuses on the racial composition of neurosurgery residencies. Over the same nine-year span, the percentage of Black residents in neurosurgery programs remained relatively constant, hovering around 5%, indicating a persistent lack of growth in representation in this specialty.

**Table 1:** Black inequalities in neurosurgery.

Authors	Summary
Affirmative Action: History and Rationale^1^	In 1955, only 4.9% of college students (18–24) were Black, but after affirmative action in the late 1960s and early 1970s, their enrollment grew (1970: 7.8%, 1980: 9.1%, 1990: 11.3%), though post-1977, their enrollment rates lagged behind those of White high school graduates.
Black Students in Higher Education^5^	By 2020, the enrollment had significantly risen, with 36% of Black individuals aged 18–24 enrolled in college.
Arcidiacono et al, (2019)	Research indicates that White applicants to a certain institution have an evident advantage if they belong to the ALDC category, an abbreviation for Alumni (legacies), Dean's List, Athletes, or Children of faculty. A White applicant with no ALDC ties typically faces a 10% chance of admission. However, their odds significantly increase if they have any of the ALDC connections. If they are legacies, their chances are five times better than the standard 10%. If they are on the dean's special list, their probability surpasses seven times that baseline rate. And if they are sought-after athletes, they're almost assured an admission. Yet, interestingly, when looking at those who were admitted because of their ALDC status, only one-fourth of them would have been admitted if evaluated without the ALDC advantage.
Bhutta et al, (2020)	The 2019 Survey of Consumer Finances (SCF) indicates persistent wealth disparities among racial and ethnic groups, consistent with 2016 findings. Specifically, the median wealth of White families is approximately eight times that of Black families.
Maqsood et al, (2021)	The US Census Bureau data shows that minority groups are experiencing rapid population growth, with half of American children under 18 being from ethnic minorities. From 1990 to 2015, the number of foreign-born residents more than doubled, accounting for a third of the total population growth.
Gabriel et al, (2021)	By 2044, racial minorities will become the majority in the U.S., and there's a growing emphasis on promoting diversity in the medical field, covering aspects of gender, race, and ethnicity.
Persad-Paisley et al, (2022)	Between 2012 and 2020, Black medical students applied to neurosurgery residency programs at a rate of 0.4 per 100,000 Black residents in the US. During this period, while the application rate for Black students decreased, it remained steady for White students.
Barrie et al, (2022)	Despite Black medical graduates showing a heightened interest in neurosurgery, they represent less than 4% of neurosurgery trainees, indicating a disparity in minority representation in the field.
Kabangu et al, (2023)	The match rate for Black applicants is notably lower than for non-Black applicants (*P* < 0.001).
Chares et al, (2023)	In 2019, only 4.95% of neurosurgical residents in the U.S. were Black. However, in 2020, the Black Lives Matter movement prompted universities to emphasize fairness, given that diverse medical groups lead to better patient outcomes and research advancements.
Hamilton et al, (2023)	On October 31, 2022, the Supreme Court discussed the use of race-based considerations in undergraduate admissions at both Harvard University and the University of North Carolina. The Court's decisions on these issues could significantly limit or entirely remove the role of race in admission choices. Evidence suggests that medical students from diverse educational backgrounds tend to be better equipped to treat patients from various backgrounds.
Do Black patients fare better with Black doctors?^8^	Patients are often more satisfied and adherent to medical advice when treated by doctors of their own race, impacting the healthcare outcomes of Black individuals.
Fact Sheet: The Current Administration Announces Actions to Promote Educational Opportunity and Diversity in Colleges and Universities^10^	After the end of affirmative action, the current administration is focusing on clarifying lawful admissions practices, providing resources to colleges, supporting underserved communities, convening educational discussions, and exploring strategies to increase diversity and address current admissions pitfalls.
Duff-Brown, (2018)	African-American doctors significantly increase the likelihood of Black men seeking preventive healthcare services, potentially reducing cardiovascular mortality in this demographic by 19%. This underscores the importance of racial diversity among physicians and its impact on health outcomes in African-American communities.
Nelson et al, (2022)	Persistent racial disparities in healthcare are influenced by factors like provider bias and systemic inequities, result in lower standard care for Black Americans compared to Whites, affecting various aspects of healthcare including disease management and patient-provider relationships.
William et al, (2019)	The article outlines how racism, as a systemic and multifaceted social issue, detrimentally impacts health outcomes and exacerbates ethnic health disparities through various mechanisms at structural, cultural, and individual levels.
Braveman et al, (2015)	Socioeconomic factors play a complex role in the racial disparity observed in preterm births between Black and White women, with no significant differences in the most disadvantaged groups but persistent disparities among more advantaged subgroups. This suggests that factors like chronic stress and unmeasured dimensions of socioeconomic disadvantage, particularly affecting Black women, may contribute to this disparity.
Firebaugh et al, (2016)	The study shows a decrease in neighborhood poverty disparity among Black Americans from 1980 to 2010 compared to other racial groups, but highlights that Black Americans still face higher overall rates of poverty.
Lee et al, (2018)	The main idea of the research is that discrimination exposure, particularly in predominantly White neighborhoods, leads to altered cortisol levels in African American emerging adults, indicating a link between neighborhood racial composition and stress-related health impacts.

Moreover, Table 1 provides a comprehensive overview of studies examining inequalities faced by Black medical students. Persad-Paisley et al 2022 observed that from 2012 to 2020, applications to neurosurgery programs from Black medical students decreased, while the rates from White students remained unchanged.^18^ Building on this, Barrie et al 2022 underscore a pronounced disparity: while Black medical graduates frequently express interest in specialized fields like neurosurgery, their actual representation in these programs is starkly lower. This suggests potential systemic barriers hindering minority students in medicine.^3^ Furthering this narrative, Kabangu et al 2023 reported that Black medical school applicants that are applying for residency experience a lower match rate in neurosurgery compared to their non-Black counterparts.^14^ Addressing a broader context, Charles et al 2023 noted that even though the percentage of Black neurosurgical residents in the U.S. was minimal in 2019, the subsequent rise of the Black Lives Matter movement in 2020 influenced universities to prioritize diversity. This shift is rooted in the understanding that diverse medical teams enhance both patient care and research.^7^

In a 2023 study by Hamilton and colleagues, they delved into the Supreme Court's examination of race-conscious admissions policies at notable institutions like Harvard and the University of North Carolina back in October 2022. The Court's potential rulings at that time were seen as jeopardizing these policies, with broader ramifications for diversity across different sectors.^13^ Following this examination, the Supreme Court, by June 2023, concluded to terminate Affirmative Action.

The next articles in Table 1 discuss the benefits of diversity and strategies to further diversify the educational system. The paper, titled “Do Black patients fare better with Black doctors?“, highlights the influence of racial concordance between doctors and patients on patient satisfaction, comprehension, adherence to medical advice, and overall health outcomes.^8^ For instance, in 2018, Duff-Brown discusses how African American doctors can reduce cardiovascular mortality among Black men by 19% and that Black patients are 29% more inclined to discuss health concerns and consent to advanced screenings when treated by Black doctors.^9^ Moreover, in a 2022 study by Nelson and colleague, the findings of the Kaiser Family Foundation's survey reveals a perception gap regarding racial bias in healthcare. While 29% of physicians acknowledge the existence of racial bias, the general population perceives race as affecting health care at a rate of 47%. This discrepancy is stark among White and Black physicians, with a mere 4% of White physicians recognizing frequent racial bias compared to 41% of their Black physicians. The consequences of this limited racial diversity in healthcare professions are significant and profound.^17^

Building on these insights the concluding article, FACT SHEET: The Current Administration Announces Actions to Promote Educational Opportunity and Diversity in Colleges and Universities, details how the White House is striving to ensure lawful, diverse, and inclusive college admissions practices.^10^ The White House's Administration is doing this by, including lawful admissions practices and valuing students' resilience in the face of adversity, aiming to sustain a diverse workforce. Such diversity is essential for culturally competent care in health care and improves health outcomes by ensuring that healthcare providers can effectively address the varied needs of all patient populations.

The persistence of a racial gap in healthcare perceptions and practices can be further compounded by systemic racism, leading to discrimination and bias that negatively impact patient experiences and health outcomes. William et al, (2019) in Table 1 found that the critical cultural competence that racial concordance provides goes beyond a mere soft skill; it becomes a significant determinant of health outcomes. The lack of diversity among healthcare providers not only restricts the scope for culturally sensitive care but also perpetuates the structural barriers that foster health disparities. Implicit biases in clinical settings can lead to substandard medical care and poorer communication, while stereotype threats and internalized racism may degrade patient trust and adherence to medical advice, worsening health outcomes. It is imperative to enhance diversity in the medical workforce to counteract the entrenched biases and systemic obstacles that compromise minority health.^19^

The recent literature on socioeconomic barriers and disparities faced by Black Americans highlights several critical issues. Research conducted in 2015 by Braveman and colleagues draws attention to the persistently higher rates of preterm birth among Black populations compared to White Americans. This disparity is not just a medical concern but is deeply intertwined with broader socioeconomic factors. Factors such as income, wealth, education, and neighborhood characteristics - including poverty rates, unemployment, segregation, and crime - are identified as significant contributors to this health issue, underscoring the complex interplay between socio-economic status and health outcomes.^6^ Additionally, in a 2016 study by Firebaugh et al, the focus shifts to the spatial dimensions of racial disparities. Their findings reveal that in metropolitan areas, Black residents are disproportionately likely to live in neighborhoods with extreme poverty, defined as areas where the poverty rate exceeds 40%. This concentration in high-poverty neighborhoods has far-reaching consequences, limiting access to quality education, healthcare, employment opportunities, and robust social networks, all of which are crucial for economic and social mobility.^11^Furthermore, the research by Lee and colleagues adds another dimension to this discussion by examining the physiological impacts of these socioeconomic disparities. They report that Black individuals living in predominantly White communities exhibit higher levels of cortisol, a stress hormone. This finding is significant because it links the experience of living in a racially incongruent community to tangible health impacts, suggesting that the stress of such environments may contribute to the overall health disparities observed in the Black population.^15^

**Fig. 5:**
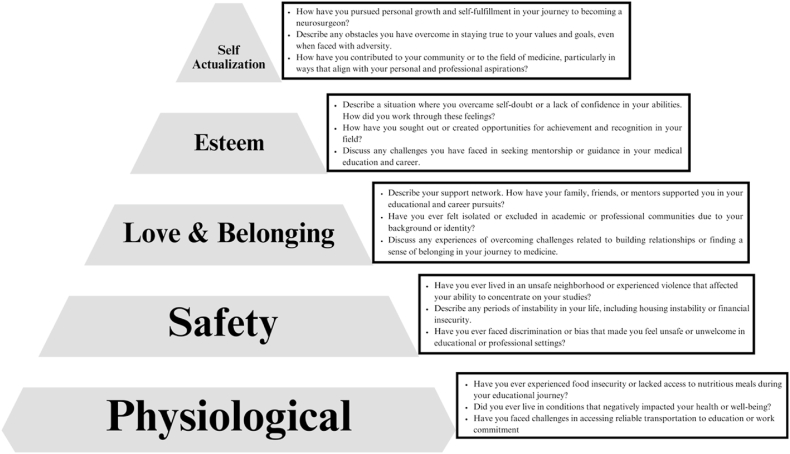
Assessment of Neurosurgery Candidates Based on Maslow's Hierarchy of Needs This model illustrates a tiered assessment framework based on Maslow's Hierarchy of Needs, designed to evaluate neurosurgery residency applicants on personal growth, community contribution, and adversities faced.

The prevailing research highlights the complex interplay of racial disparities within the United States, delineating a web of socio-economic, spatial, and physiological elements that shape the health and welfare of Black communities. Fig. 4, Fig. 5 integrates MAI with Maslow Hierarchy of Needs to guide Neurosurgery Residency Interview Committees. This framework helps in recognizing the disparities that disproportionately impact marginalized groups. According to Maslow's theory, satisfying fundamental needs is essential before addressing higher-level aspirations. The hierarchy is structured from the base upwards, including physiological, safety, love and belonging, esteem, and self-actualization needs.

**Fig. 6:**
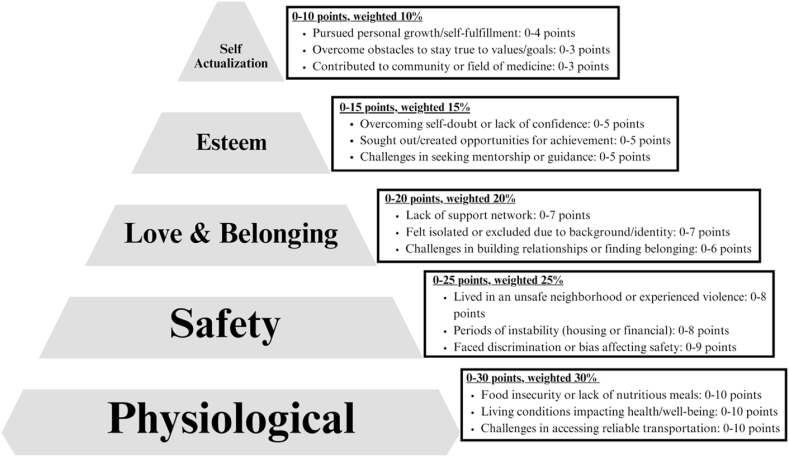
Point-Based Assessment Criteria for Neurosurgery Residency Applicants Across Maslow's Hierarchy Outlines a structured scoring system, assigning weighted points to each level of Maslow's hierarchy, to objectively quantify the diverse challenges encountered by neurosurgery residency candidates.

**Fig. 7:**
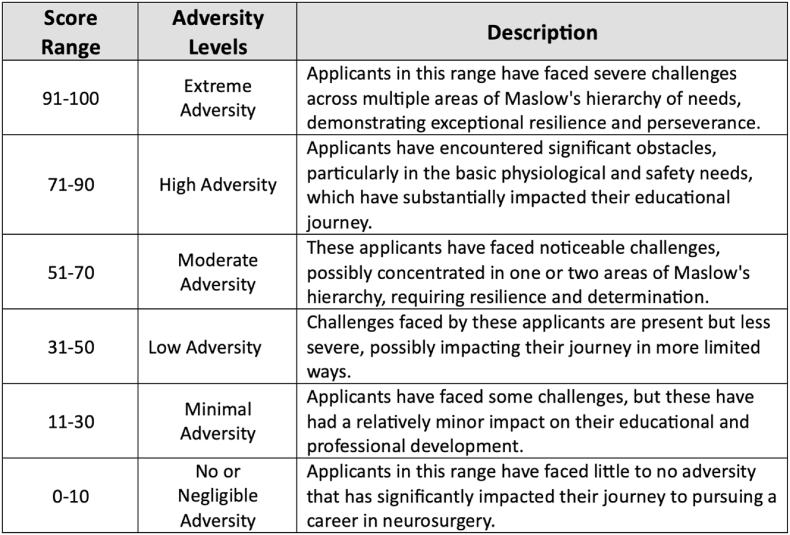
Adversity Score Classification for Neurosurgery Residency Applicants This table categorizes applicants based on adversity scores, detailing the extent of challenges faced within Maslow's hierarchy of needs, from no adversity to extreme adversity, to provide a nuanced perspective on each candidate's journey.

The NEURO-ASCEND (Neurological Applicant Scoring Criteria Embracing Neurosurgery Diversity) Framework, which is part of the broader MAI (Fig. 1), can be utilized by residency committees by formulating questions to delve into each tier of needs during interviews, as illustrated in Fig. 5. Moreover, a weighted score is attributed to each level, reflecting its importance in the individual's development, as depicted in Fig. 6. This methodology empowers committees to quantify adversity through a cumulative score, which is demonstrated in Fig. 7, thereby enabling a more equitable evaluation of candidates from diverse backgrounds."

## 4 Discussion

The United States holds the distinction of being the world's third most populous nation. Intriguingly, recent data suggests rapid demographic shifts, with ethnic minority children now representing half of the under-18 population.^16^ In this context, the influence of Affirmative Action policies in expanding educational access for groups that have historically faced marginalization becomes critical to explore. This impact is vividly illustrated by the significant uptick in college enrollments among Black students. As a testament to these policies, there has been a transformative increase in their college attendance rates: starting from 4.9% in 1955, to 36% by 2020 as seen in Figs. 2^1,5^. While college attendance among Black students has risen since the implementation of affirmative action in the 1960s, they still trail behind their White counterparts, particularly in specialized fields where challenges persist. For instance, over the last decade, efforts to achieve equitable representation in academic neurosurgery have seen only moderate success.^3^ African-Americans remain underrepresented, making up less than 4% of neurosurgery training programs.^3^ Interestingly, data indicates that Black medical students in 2012 were 18% more likely to apply to neurosurgery residencies than their White counterparts, though their absolute numbers remain low.^18^ Given the projected demographic shift by 2044, where racial minorities are anticipated to become the majority, addressing these academic disparities is of paramount importance.^12^

The cessation of Affirmative Action policies poses a significant threat to the already fragile representation of diverse backgrounds in medical institutions. Such a step could have far-reaching consequences, detrimentally affecting Black pre-medical students and amplifying health disparities for communities of color across the nation.^13^ Additionally, Fig. 3 shows that between 2012 and 2018, there was a notable decline in the number of Black medical students applying for neurosurgery residencies, whereas the rate for White medical students remained disproportionately much higher.^18^ Over the past nine years, even with more residency slots created, the percentage of Black neurosurgery residents has not increased, suggesting persistent systemic barriers to racial equity, as shown in Fig. 4^12,^ . Without Affirmative Action in place, these systemic biases may intensify, widening the gap in university admissions**.** This decline in university admissions for Black students, exacerbated by the absence of Affirmative Action, could directly contributes to the dwindling pool of Black applicants for neurosurgery residencies, further aggravating the discrepancy in match rates. Such a trend not only diminishes the diversity in the neurosurgical workforce but also indirectly leads to inferior patient care outcomes, as diverse medical teams have been shown to better understand and address the unique needs of a multicultural patient base.

The lawsuit, “Students For Fair Admissions v. Harvard University,” offers insightful revelations about the pivotal role of Affirmative Action in university admissions. The lawsuit illuminated the embedded biases in Harvard's admissions criteria. It was discovered that ALDCs received a disproportionate advantage. Over 43% of the admitted White students fell into one of these ALDC categories, which is in stark contrast to less than 16% of admitted African American, Asian American, and Hispanic students.^2^ Furthermore, studies have indicated that removing preferences for athletes and legacies would lead to a marked change in the racial makeup of the admitted cohort. The share of White students would dwindle, while figures for other racial groups would either ascend or stay consistent.^13^

These findings underscore the importance of considering factors like Affirmative Action in promoting diversity in academia. Such policies aim to provide opportunities for traditionally marginalized racial and ethnic communities to access prestigious institutions and specialized programs like neurosurgery. It is important to acknowledge the significant underrepresentation of Black individuals among practicing neurosurgeons, a phenomenon that may be influenced, at least in part, by the disproportionate impact of the social determinants of health on access to medical education and training. This imbalance becomes even more apparent when we consider that the average White family possesses approximately eight times the wealth of a typical Black family.^4^

Significant financial disparities present substantial challenges for aspiring doctors from economically disadvantaged backgrounds, and Black individuals often encounter particularly formidable obstacles. These challenges can begin early in life, with Black infants experiencing higher rates of mortality, lower birth weights, and preterm births.^6^ Moreover, Black Americans are more likely than their counterparts to reside in poverty-stricken neighborhoods, where they face inadequate access to quality education, healthcare, job opportunities, and social networks.^11^ Systemic racism has also been associated with increased cortisol levels in African Americans, further highlighting the enduring effects of these disparities. It is important to note that these facts do not imply racial inferiority but rather point to systemic issues that need to be addressed.^15^

The enduring disparities in starting points, affecting individuals from various backgrounds, continue to shape their life trajectories, resulting in an uneven playing field that presents greater challenges for those aspiring to pursue a career in neurosurgery. The hurdles faced by medical students, regardless of their racial background, extend into critical aspects of neurosurgical training. Affordability becomes a concern when considering away rotations and the associated living expenses, which are crucial for gaining exposure to the field. Additionally, the cost of traveling for interviews can be a significant barrier. These economic challenges are exacerbated for students who may seek to enhance their residency applications with a research gap year, a strategy often out of reach for those without adequate financial support.

Maslow's Hierarchy of Needs offers a valuable framework for understanding how these disparities impact the journeys of aspiring neurosurgeons. A medical student in this context may be contending with unmet physiological and safety needs, while also grappling with issues such as food insecurity, living in disadvantaged neighborhoods, facing transportation challenges, and coping with discrimination. They may not have the support of a loving family and may even be using their student loans to provide for their own families. In contrast, a student from a more privileged background may not face the same physiological needs, enjoying food security, residing in a more favorable neighborhood, and benefiting from a supportive family that can provide financial assistance for study materials and access to additional resources for exams.

To address these disparities and foster diversity in the field of neurosurgery, our team has devised a point-based assessment model for Neurosurgery Residency Committees to utilize during candidate evaluations and interviews. As depicted in Fig. 5, this model involves querying candidates about their fulfillment across various levels of Maslow's Hierarchy of Needs, commencing with physiological and safety needs and progressing through love and belonging, esteem, and self-actualization. Fig. 6 illustrates the alignment of each hierarchy level with a set of questions, weighted using MAI, where point allocations range up to 100. Fig. 7 underscores that a higher Maslow score serves as an indicator prompting the admission committee to consider the candidate's life circumstances and the challenges they have encountered when rendering their judgment. This approach seeks to level the playing field and provide equitable opportunities for diverse students aspiring to pursue careers in fields like neurosurgery.

The validation of MAI in both single and multicenter studies is proposed in Fig. 1. This initiative aims to develop the NEURO-ASCEND (Neurological Applicant Scoring Criteria Embracing Neurosurgery Diversity) Framework, a potential tool for establishing an objective measurement system that could supplant the need for affirmative action in fostering equity. It is imperative for faculty, staff, and the wider scientific community to endorse a holistic approach in the assessment of candidates during admissions processes. Admissions committees should broaden their evaluation criteria beyond traditional metrics such as grades and standardized test scores, placing greater emphasis on personal attributes and the challenges applicants have overcome.^13^

This expanded approach necessitates an acknowledgment of prevalent health disparities and societal inequities. For example, consideration of an applicant's ZIP code could be integral, as those from economically disadvantaged areas often confront more significant educational, environmental, and health-related hurdles than those from affluent locales. Additional factors worth considering might include an applicant's dependence on public aid programs like the Supplemental Nutrition Assistance Program or Medicaid/Medicare, or their uninsured status. This enriched perspective reshapes the conventional understanding of ‘excellence'.^13^ While these strategies do not directly replace Affirmative Action, they pave a novel pathway towards preserving diversity and broadening the scope of excellence in higher education. Recognizing and valuing these socioeconomic barriers is a crucial step in further diversifying the academic landscape.

Additionally, the limitation of not prioritizing diversity within the healthcare system could lead to suboptimal patient care and satisfaction. Studies have shown that patients tend to give higher ratings to physicians of the same racial or ethnic background.^13^ Furthermore, research indicates that Black patients often feel more comfortable discussing health concerns and are more likely to consent to advanced screenings when cared for by Black physicians.^9^ This enhanced willingness to engage in healthcare discussions and procedures may stem from shared cultural experiences and a deeper sense of relatability between the patient and the doctor. In contrast, while 29% of doctors acknowledge the existence of racial bias in healthcare, patients perceive the influence of race on health outcomes at a higher rate.^17^ Implicit biases in healthcare, which may not always be overtly recognized by providers, can lead to inferior medical care and impede effective communication. This discrepancy suggests that patients might be hesitant to fully engage or feel judged in healthcare settings that lack racial and cultural representation, potentially leading to a reluctance in sharing vital health information or needs and perpetuating the cycle of health care disparities.^19^ In counties where there are fewer primary care doctors who are Black, the life expectancy of Black inhabitants may not be as prolonged as in areas with more Black primary care doctors.^8^ A lack of diversity in medical school student bodies might limit the comfort and effectiveness of future physicians when treating diverse patient demographics.^13^

In light of the recent Supreme Court verdict abolishing Affirmative Action in higher education, it is imperative that a new course be charted by the White House that integrates consideration of adversity into the admissions processes of higher education institutions. This forward-thinking method is aimed at providing guidance on legally permissible ways to sustain a diverse student body. In support of this vision, a National Summit on Educational Opportunity is to be launched.^10^

To fortify this initiative, the Department of Education, in collaboration with the Department of Justice, is preparing a comprehensive report that will outline best practices and policy guideline.

This report will pinpoint strategies to boost diversity and enrich educational opportunities in tertiary institutions. At the heart of these strategies is the intent to thoroughly weave adversity considerations into the admissions matrix. Moreover, there is a push for greater transparency in college admissions and enrollment procedures. The overarching objective is for states to be enabled to harness data in crafting programs that effectively reach out to historically underrepresented group. It is essential for these processes to reflect upon socioeconomic challenges, especially in light of the persistent racial and ethnic wealth divides in the United States.^10^

In a nation where demographic shifts highlight a burgeoning ethnic minority, the decisions that shape our educational landscape have never been more critical. The abolishment of Affirmative Action has set into motion a series of contemplative ripples, urging stakeholders to evaluate not only the spirit of diversity but also its tangible effects in our institutions. The intricacies of these decisions extend beyond mere enrollment, reaching into the very heart of the medical world where representation can directly influence patient care, satisfaction, and even life expectancy. As America stands at this educational and societal crossroads, it becomes evident that diversity isn't just a policy checkbox but a critical facet of our shared progress. The steps taken by administrations, institutions, and residency programs in weaving adversity considerations, championing holistic methodologies, and reshaping traditional notions of excellence, will signal a commitment to a more inclusive, comprehensive, and equitable future. While the tools may evolve, the enduring goal remains unchanged: creating an academic and professional realm where every background finds voice, representation, and opportunity.

## CRediT authorship contribution statement

**Albert Alan:** Writing – review & editing, Writing – original draft, Visualization, Validation, Resources, Project administration, Methodology, Investigation, Formal analysis, Data curation. **Michelle Ennabe:** Writing – review & editing, Writing – original draft, Visualization, Validation, Methodology, Investigation, Formal analysis, Data curation, Conceptualization. **Abdulmuizz Sulaiman:** Methodology, Formal analysis, Data curation. **Martin Weinand:** Writing – review & editing, Visualization, Methodology, Data curation, Conceptualization.

## Declaration of competing interest

The authors declare that they have no known competing financial interests or personal relationships that could have appeared to influence the work reported in this paper.

## Abbreviations List

ALDC: Alumni (legacies),Dean's List, Athletes or Children of Faculty
SCF: Survey of Consumer Finance
MAI: Maslow Adversity Index
NEURO-ASCEND: Neurological Applicant Scoring Criteria Embracing Neurosurgery Diversity
